# When Less Is More: Specific Capture and Analysis of Tumor Exosomes in Plasma Increases the Sensitivity of Liquid Biopsy for Comprehensive Detection of Multiple Androgen Receptor Phenotypes in Advanced Prostate Cancer Patients

**DOI:** 10.3390/biomedicines8050131

**Published:** 2020-05-22

**Authors:** Chiara Foroni, Natasa Zarovni, Laura Bianciardi, Simona Bernardi, Luca Triggiani, Davide Zocco, Marta Venturella, Antonio Chiesi, Francesca Valcamonico, Alfredo Berruti

**Affiliations:** 1 CREA Laboratory (Centro di Ricerca Emato-Oncologica AIL), ASST Spedali Civili of Brescia, 25123 Brescia, Italy; simona.bernardi86@gmail.com; 2 Oncology Unit, Department of Medical and Surgical Specialties, Radiological Sciences and Public Health, University of Brescia, ASST Spedali Civili of Brescia, 25123 Brescia, Italy; franzval@yahoo.it (F.V.); alfredo.berruti@unibs.it (A.B.); 3 Exosomics S.p.A Siena, Strada del Petriccio e Belriguardo 35, 53100 Siena, Italy; lbianciardi@exosomics.eu (L.B.); dzocco@exosomics.eu (D.Z.); mventurella@exosomics.eu (M.V.); achiesi@exosomics.eu (A.C.); 4 Unit of Blood Diseases and Stem Cell Transplantation, Department of Clinical and Experimental Sciences, University of Brescia, ASST Spedali Civili of Brescia, 25123 Brescia, Italy; 5 Radiation Oncology Department, University of Brescia, ASST Spedali Civili, 25123 Brescia, Italy; luca.triggiani@unibs.it

**Keywords:** exosomes, extracellular vesicles, castration resistant prostate cancer, *AR*-V7, liquid biopsy

## Abstract

We evaluated the advantages and the reliability of novel protocols for the enrichment of tumor extracellular vesicles (EVs), enabling a blood-based test for the noninvasive parallel profiling of multiple androgen receptor (*AR)* gene alterations. Three clinically relevant *AR* variants related to response/resistance to standard-of-care treatments (*AR*-V7 transcript, *AR* T878A point mutation and *AR* gene amplification) were evaluated by digital PCR in 15 samples from patients affected by Castration-Resistant Prostate Cancer (CRPC). Plasma was processed to obtain circulating RNA and DNA using protocols based on tumor EVs enrichment through immuno-affinity and peptide-affinity compared to generic extraction kits. Our results showed that immuno-affinity enrichment prior to RNA extraction clearly outperforms the generic isolation method in the detection of *AR*-V7, also allowing for a distinction between responder (R) and non-responder (NR) patients. The T878A mutation was detected, overall, in nine out of 15 samples and no approach alone was able to reveal mutations in all harboring samples, showing that the employed methods complement each other. *AR* amplification was detected in the majority of CRPC samples analysed using either cell-free DNA (cfDNA) or exosome isolation kits (80%). We demonstrated that selective isolation of a subset of circulating exosomes enriched for tumor origin, rather than analysis of total plasma exosomes, or total plasma nucleic acids, increases sensitivity and specificity for the detection of specific alterations.

## 1. Introduction

In recent years, the therapeutic landscape of Prostate Cancer (PCa), in particular for patients with advanced and Castration-Resistant Prostate Cancer (CRPC), has rapidly expanded with the introduction of several new approved drugs that represent the second generation of anti-androgens promising remarkable survival benefits. Regrettably, the blind and unoptimized use of anti-androgen therapies, in an inappropriate patient selection scenario had led to the poor overall efficiency of treatment, elevated costs, a loss of time and reduced patient survival and quality of life [1].

Many preclinical and epidemiologic data corroborate the central role of androgen receptor (*AR*) signaling in Prostate Cancer (PCa) oncogenesis and disease progression. *AR* gene aberrations are rare in primary tumors before exposure to hormone therapy, but occur in over 60% of patients with metastatic disease [2]. The amplification or mutation of the *AR* gene [3] and the expression of truncated *AR* splice variants that display ligand-independent activity [4] have been linked to molecular mechanisms of resistance/response not only to anti-androgen drugs (such as abiraterone and enzalutamide), but also to other standard-of-care treatments for advanced PCa (such as taxols) [5]. Moreover, the body of evidence documents the preliminary efficacy of AR signaling inhibitors in other malignancies such as breast cancer, bladder cancer, kidney cancer, pancreatic cancer, hepatocellular cancer, ovarian and endometrial cancers. Therefore, accurate and serialized profiling of the ample spectrum of *AR* mutations with high specificity and sensitivity emerges as crucial for a dynamical and readily monitoring of the therapeutic resistance and progression of the disease.

Minimally invasive blood-based “liquid” biopsies provide an attractive and practical tool to track a patient’s response, or as a “surveillance” method for people who have completed treatment. The development and validation of this type of biomarker-based assays, addressing the status of clinically relevant biomarkers (i.e., AR), would positively affect the management of PCa patients. However, there still exist important challenges that affect the application of liquid biopsies in clinical practice.

Circulating tumor DNA (ctDNA) is a very small fraction of cell-free DNA (cfDNA) that cannot be selectively isolated and distinguished from a large background of non-tumor derived circulating DNA. Moreover, it is confirmed that the major sources of circulating DNA fragments, either complexed with nucleo- or lipoproteins or naked, are apoptosis or necrosis, making them less representative of living and proliferating cells [6]. The availability of free circulating transcripts, in particular, longer mRNA fragments in blood, is extremely limited, although these might not be as fragile as previously assumed. Freely circulating RNA in plasma is reported to be protected within vesicular structures [7] or bound to DNA in nucleosomes or DNA–lipoprotein complexes [8]. Circulating tumor cells (CTCs), instead, are ideally representative of parental tumors but tend to be late and exceedingly rare events whose detection requires expensive devices and cumbersome operations [9].

Circulating extracellular vesicles (EVs) offer a new liquid biopsy approach that could overcome drawbacks related to the use of CTCs or circulating nucleic acids (CNA), mostly ctDNA. These vesicles, which are exceptionally stable, cluster preserved and functionally relevant tissue and disease markers (proteins and nucleic acids) and are accessible in biofluids with minimally or noninvasive procedures. Exosomes are a nanosized subclass of EVs, originating from the endosomal cell compartment, that are found to be abundant in biofluids such as blood and urine, and have been extensively studied as biomarker reservoirs. Despite promising features [10], exosome-based tests are not yet considered clinical grade. The first exosome-based laboratory diagnostic test, EPI (Intelliscore, Exosome Diagnostics, Inc.), has been recently approved for medical insurance coverage in the US as a prognostic test for the distinction of indolent from clinically significant PCa in conjunction with standard-of-care procedures, while no FDA-approved EV-based IVD assays or biomarkers exist today.

A common feature of recently reported studies exploiting plasma-derived exosomes is that the biomarkers are isolated from the bulk of circulating exosomes and not from a specific subpopulation of cancer cell-derived vesicles, thus limiting the effectiveness and advantage of the exosome-based approach [11].

With this study, we investigated the feasibility of novel protocols for the enrichment of tumor EVs that employ specific affinity-mediated selection platforms. The objective is to develop a robust and clinically feasible blood-based test which enables the noninvasive parallel profiling of multiple *AR* alterations in relation to response/resistance to standard-of-care treatments for CRPC. In a pilot study on a small cohort of 15 patients with CRPC, we concomitantly assessed three *AR* gene variants falling into different biomarker categories, namely a somatic point mutation T878A, a copy number variation in the *AR* gene as an indication of gene amplification, and levels of expression of full-length *AR* and AR-V7 splice variants.

The aim of the present study was to explore the feasibility and the advantage of a tumor exosome-based approach to support the accurate and timely stratification of CRPC patients in the context of their response to antiandrogen therapy. The results were evaluated based on the patients’ classification according to conventional clinicopathological criteria and on the determination of the same genomic variations in free CNA extracted from the same blood samples.

## 2. Materials and Methods

### 2.1. Patient Selection and Classification

The study sample was 13 patients with metastatic CRPC (Table 1); all were receiving therapy at the time of sample collection. Previous anticancer therapies were also recorded. The study was approved by the Ethics Committee of Brescia, Italy (local study no. NP2813 approved in July 2017), and conducted in accordance with the principles of the Declaration of Helsinki. Written, informed consent was obtained before blood collection and analysis. Patients were monitored according to a standard surveillance timeline and classified as responders (R) and non-responders (NR) to therapy by oncologists who evaluated instrumental and/or biochemical parameters during treatment. Eight patients were classified as NRs to treatment, three as Rs. The status of two patients from which two plasma aliquots were collected at different timepoints changed from an R (samples 2 and 3), to a NR (samples 11 and 12, respectively). The samples from these two patients were analyzed independently, bringing the overall sample size to 15 samples.

### 2.2. Plasma Collection and Nucleic Acid Isolation

Peripheral blood was collected in 4.9 mL K2EDTA tubes and processed within 30 min after drawing. Plasma was obtained by centrifuging at 2000× *g* for 10 min at room temperature and then stored at −80 °C until analysis. RNA from plasma samples was extracted using four different protocols: (1) a SoRTEV™ RNA Enrichment Kit (Exosomics SpA, Siena, Italy), providing tumor EVs enrichment by proprietary immuno-affinity (IA) selection (hereafter IA-RNA protocol). The antibody employed within the SoRTEV kit is a part of a proprietary library of antibodies (Exosomics S.p.A) raised against exosomes (nanosized EVs) derived from primary tumor cell lines. Although its target is undisclosed, this antibody has been selected during the extensive screening on tumor and non-tumor models and samples in which this SoRTEV Ab displayed preferential binding to EVs, in particular exosome-like vesicles (in terms of size and molecular content), from tumor cell lines grown in hypoxic conditions, and to exosomes from plasma samples obtained from cancer patients (Supplementary Figure S1). This specificity is not related to tissue type; the SoRTEV target is not prostate specific but rather cancer specific, as it has been observed in different cancer types such as Melanoma, Colon Cancer, Prostate Cancer and Chronic Myeloid Leukemia [12]. The SoRTEV kit was used according to its technical manual: briefly, the workflow consists of a first isolation step that is based on the incubation of immunobeads with the plasma sample; bead–exosome complexes are then pulled down by centrifugation and undergooptimized, column-based RNA extraction; (2) RNA extraction is then carried out after the peptide-affinity (PA) isolation of exosomes (hereafter PA-RNA protocol) by employing the ME™ Kit (New England Peptide, Gardner, MA, USA). This kit features a Vn96 peptide that has an affinity to Heat Shock Proteins (HSPs). Although some HSPs are considered common exosome markers, their expression is highly increased on the surface of tumor cells and there-released vesicles, with respect to vesicles coming from non-tumor cells. Thus, Vn96 provides a stronger affinity capture of HSP-overexpressed exosomes from a given body fluid, which is claimed to provide superior diagnostic value for cancer diagnoses [13]. This step of incubation with the ME^TM^ kit, according to the manifacturer’s instructions, was followed by Trizol-based RNA purification; (3) moreover, the exoRNeasy serum/plasma kit (Qiagen, Venlo, The Netherlands), a more generic extraction protocol that takes advantage of membrane-affinity columns (exoEasy columns) to bind exosomes and other EVs, was used. They are afterwards lysed by Qiazol and spin column-based RNA purification is performed; (4) finally, the miRNeasy serum/plasma kit (Qiagen, Venlo, The Netherlands) was used for a subset of samples (*n* = 6, 4 from NR and 2 from R) from which leftover plasma was available; the cell-free total RNA from plasma was purified following the manufacturer’s instructions. Briefly, plasma is lysed with Qiazol and, after chloroform addition, the upper phase is purified through a silica membrane column.

The DNA from plasma was extracted by employing three methods: (1) EVs enrichment with proprietary IA selection (using the same isolation agent as the SoRTEV RNA Enrichment Kit—see above) followed by column-based DNA purification (hereafter IA-DNA protocol); (2) PA isolation of EVs with the ME™ Kit (New England Peptide, Gardner, MA, USA, see above) followed by column-based DNA purification (hereafter PA-DNA protocol); (3) a QIAamp Circulating Nucleic Acid kit (Qiagen, Venlo, The Netherlands), which purifies generic free-circulating nucleic acids from body fluids in four steps: sample lysis with the subsequent release of nucleic acids from vesicles, lipids and proteins, binding to QIAamp Mini column, washing steps, elution. For each nucleic acid extraction, 0.5 mL plasma was employed (0.2 mL for the miRNeasy serum/plasma kit); the elution volume was 15 µL for RNA and 50 µL for DNA.

### 2.3. Digital PCR (dPCR) Analysis of Relevant Targets

For each RNA sample, 12 µL of the template were retrotranscribed to cDNA using a QuantiTect Reverse Transcription Kit (Qiagen, Venlo, The Netherlands) for a final volume of 20 µL. The assessment of AR-V7, AR-FL and RNY4 copies was performed on cDNA using custom assays (primers described elsewhere) [12,14]. DNA was employed to detect the *AR* T878A variant (TaqMan™ SNP Genotyping Assay C_175239649_10, Thermo Fisher Scientific, MA, USA), *AR* gene copy numbers with RNaseP as the internal reference standard (TaqMan™ Copy Number Assay Hs04107225_cn and TaqMan™ Copy Number Reference Assay, human, RNase P, Thermo Fisher Scientific, USA). A volume of 15 μL of the reaction mix was loaded on a QuantStudio 3D Digital PCR 20K Chip (Thermo Fisher Scientific, MA, USA) and thermocycling ran at 95 °C for 8 min, 40 cycles (37 cycles for the AR-V7 assay) at 95 °C for 15 s and at 60 °C for 1 min, with a final extension step at 60 °C for 2 min. Copies/µL reaction were retrieved with QuantStudio 3D AnalysisSuite Cloud Software and copies/mL plasma were calculated by multiplying this value by the dilution factors applied during the process.

### 2.4. Statistical Analysis

The obtained results refer to the absolute target concentration expressed as copies per milliliter, as determined by dPCR QuantStudio 3D AnalysisSuite Cloud Software (v. 2.0, Thermo Fisher Scientific, MA, USA ). As our study is the first one with the simultaneous assessment of all fractions in a single sample, the number of technical replicates was limited and the analysis was done by sample set comparisons. Briefly, the one-way analysis of variance (ANOVA) and Bonferroni post-hoc tests were used to determine the statistical significance of the observed differences for group comparison. The statistical analysis was performed using Prism (GraphPad Software, La Jolla, CA, USA). The difference between groups was considered statistically significant if the *p*-value was less than 0.05.

## 3. Results

The aim of this study was to take advantage of cutting-edge protocols that enrich tumor-derived exosomes both by IA or PA methods in comparison to benchmark or more generic kits, in order to explore the feasibility and reliability of exosome-based tests to allow for the detection of *AR* alterations in CRPC.

Nucleic acids were isolated from 15 CRPC plasma samples and from a pool of healthy donors, using different sample preparation methods for the collection of either total cfDNA, total circulating EVs or the enrichment of tumor originated EVs, and afterwards tested by digital PCR for the presence of *AR* variants, known to be associated with patients’ response to therapy.

### 3.1. RNA Target—AR-V7

RNA was extracted from plasma samples using different sample preparation kits. dPCR assay was performed to detect the *AR*-V7 splicing variant, *AR*-full length (FL) (the full-length version of the transcript) and the small noncoding RNY4 as a control gene transcript known to be abundant in EVs and human plasma [15].

**Figure 1:**
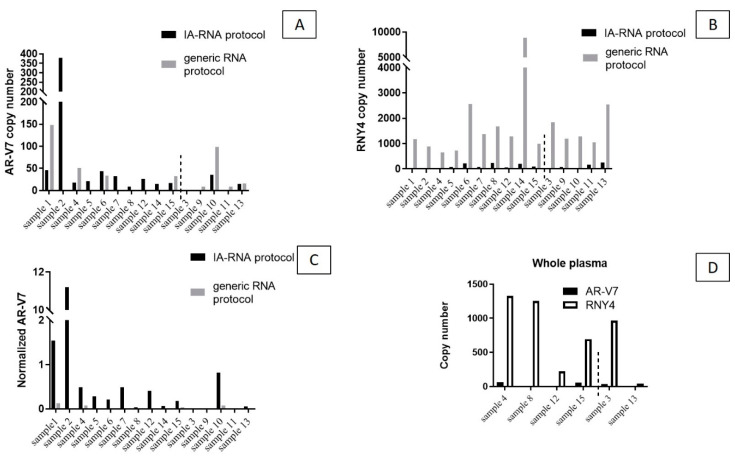
(**A**) Androgen receptor (*AR*)-V7 and (**B**) RNY4 copies/mL (respectively) detected with immune-affinity (IA)-RNA protocol and generic RNA protocol. (**C**) Normalized AR-V7 copies/mL. Note that the IA-RNA protocol isolated overall much less RNA than the exoRNeasy method. (**D**) AR-V7 and RNY4 copies/mL detected on whole plasma in a subset of samples.

The *AR*-V7 transcript (Figure 1A) was detected in 12/15 samples (80%) purified using the IA-RNA protocol (8.7–377.7 copies/mL; an average of 4.6 for all samples and 54.4 for positive ones); in 8/15 (53%) samples isolated with the generic extraction protocol (6–147.6 copies/mL; an average of 26.4 for all samples, 49.5 in *AR*-V7^+^ ones) and in 7/15 (46.6%) samples purified with the PA-RNA protocol (12.2–209.5 copies/mL; an average of 28.7 for all samples and 61.5 for positive ones).

The IA-RNA protocol distinguished NRs from Rs better than the generic extraction method. With the IA-RNA protocol, all 10 NR samples were *AR*-V7^+^ (average of 60.4 copies/mL plasma) while among the five R samples, two were positive and three were *AR*-V7^−^ (average of 10.1 copies/mL plasma).

A parallel analysis with generic extraction detected the transcript in 4/10 NR samples and in 4/5 R samples (average of 26.4 and 26.3 copies/mL, respectively). The splicing variant was detected in 5/10 NR samples and in 2/5 R samples (average of 15.4 and 55.3 copies/mL, respectively) with the PA-RNA protocol, showing an inverse correlation with patient classification in comparison to the IA-RNA method.

In terms of whole plasma, *AR*-V7 was detected in 3/6 (50%) samples, of which two were NR (average of 60.5 copies/mL) and one was R (32 copies/mL) (Figure 1D).

Figure 1B presents the data for RNY4 copies/mL. As expected, more RNY4 copies were detected by the less selective method than by protocols that enrich tumor-derived material before extraction. An average of 106.5 copies/mL were obtained with the IA-RNA protocol, 678.5 copies/mL with the PA-RNA protocol and 1869 copies/mL with the generic extraction method. An average of 109.9 copies/mL were detected in whole plasma.

RNA isolation from IA-isolated exosomes improved the detection of *AR*-V7 compared to the more generic method for EV RNA extraction. When the *AR*-V7 copies were normalized to Y4 copies, the normalized values were much higher in the samples processed with the IA protocol (Figure 1C).

**Figure 2:**
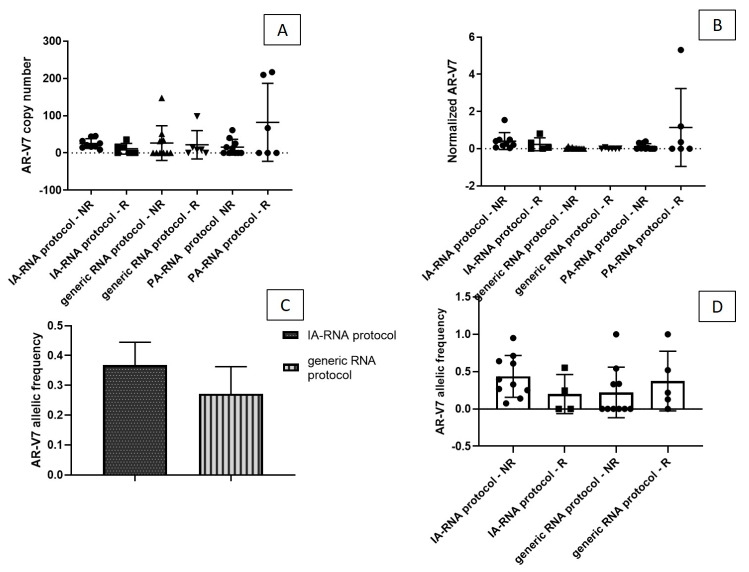
(**A**) *AR*-V7 and (**B**) normalized *AR*-V7 copies/mL, in responder (R) and non-responder (NR) samples detected with the IA-RNA protocol, the generic RNA protocol and the PA-RNA protocol. The IA-RNA protocol distinguished between R and NR patients better than the other protocols on the basis of normalized *AR*-V7 results. (**C**) The average *AR*-V7 allelic frequency detected after IA-based enrichment and generic extraction, evaluated as: *AR*-V7 copies/WT copies + *AR*-V7 copies). (**D**) The difference between the NR and the R patients for the two isolation methods.

In order to assess the potential of different sample preparation protocols to enable the correct stratification of patients in to Rs or NRs, we performed a comparative analysis. A difference between NR and R samples was observed using IA isolation. The isoform load was higher in the NR samples than in the R sample, although the difference is not statistically significant, probably because of the small sample size. No difference between the NR and the R subset was observed with the generic isolation kit, while an opposite trend was observed with the PA-RNA protocol (Figure 2A).

There was an observable, albeit not statistically significant, difference in the number of normalized *AR*-V7 copies in NR samples using the IA approach (Figure 2B).

**Figure 3:**
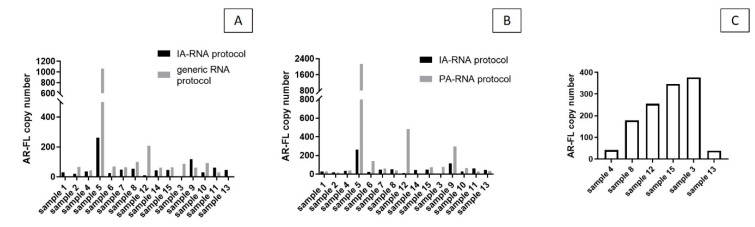
(**A**) AR-FL copies/mL identified with IA-RNA protocol vs. generic protocol, (**B**) with IA-RNA protocol vs. PA-RNA protocol and (**C**) in whole plasma in a subset of samples.

There was an increase in *AR*-V7 allelic frequency following the IA-RNA protocol (average of 36%) compared to the benchmark generic kit (average of 27%—Figure 2C). There was an average AR-V7 allelic frequency of 45.4% in the NR samples and 20% in the R samples after IA isolation and average values of 22% and 37.2% were detected in the NR and the R samples, respectively, after generic extraction (Figure 2D).The detection of *AR*-FL showed the same trend observed for *AR*-V7; an average of 55.5 copies/mL were detected with the IA-RNA protocol, 235 copies/mL with the PA-RNA protocol, and 133.5 copies/mL with the generic kit; an average of 206.2 copies/mL were detected in whole plasma (Figure 3A–C, respectively).

### 3.2. DNA Targets—AR T878A and AR Amplification

DNA was extracted from patient plasma using three sample preparation kits; *AR* T878A and *AR* gene amplification were then analyzed by dPCR (Thermo Fisher Scientific, USA).

**Figure 4:**
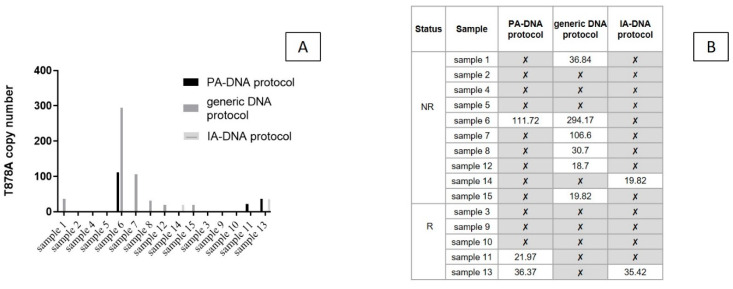
(**A**) *AR* T878A copies/mL identified with the column-based DNA purification (PA-DNA) protocol, the generic DNA protocol Kit and the IA-A protocol. (**B**) Results of the isolation for detection (with mutation copies/mL plasma)/non detection (✘) of the point mutation.

T878A point mutation was detected in 3/15 samples (20%, 22–111.7 copies/mL) using the PA-DNA protocol and in 6/15 samples (40%) after generic extraction (18.7–294.2 copies/mL) (Figure 4A). All positive samples were classified as NRs. Using the IA-DNA protocol, 2/15 samples (13%) were *AR* T878A+ (19.8 and 35.4 copies/mL, respectively) (Figure 4B).

To analyze *AR* gene amplification, RNaseP, a gene that is not known to be overexpressed in CRPC, was employed as an internal reference standard to normalize *AR* gene copies. The ratio between *AR* and RNaseP copies was calculated for each sample. The ratio of each disease sample was then normalized to the ratio of a healthy pool of male individuals. A cutoff of >1.5 was considered for gene amplification, as previously described [16].

**Figure 5:**
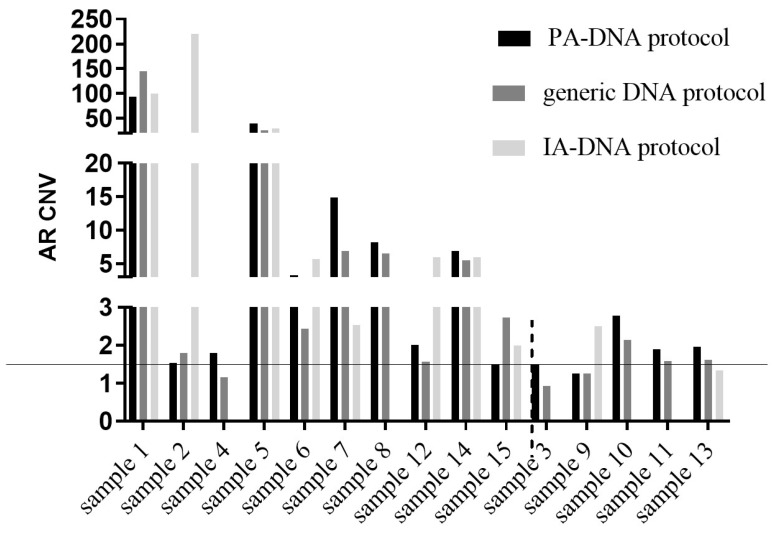
*AR* gene amplification evaluated based on the ratio between *AR* and RNaseP copies as internal controls (as previously described in [16]). The threshold for positiveness was set at 1.5.

AR amplification was detectable with the PA-DNA protocol and the QIAamp Circulating Nucleic Acid extraction kit in 12/15 samples (80%). The PA-DNA protocol returned borderline values (1.49 and 1.48) for two of the three negative samples (samples 3 and 15); the IA-DNA protocol returned positive results in 8/11 (72.7%) samples (data for four samples were not available because of technical problems) (Figure 5).

## 4. Discussion

The detection of tumor markers in both research and diagnostic settings has historically relied on the direct analysis of tumor tissue samples. However, tumor tissue biopsies collected at diagnosis or local surgery do not capture tumor clonal evolution; in addition, biopsies are associated with substantial intra-patient heterogeneity and discomfort, risks and costs. On the other hand, liquid biopsy, using the tumor material shed in circulation in the form of free CNAs, or encapsulated within CTCs or EVs, promises to provide a minimally invasive platform for the routine sampling and monitoring of advanced cancer patients.

EVs, exosomes in particular, carry tumor-derived nucleic acids, proteins, metabolites and lipids in the peripheral circulation.

In this study, we show the advantage of novel methods for enriching tumor-derived exosomes from plasma, over conventional and more generic methods for the recovery of overall EVs or overall circulating cfDNA. We have applied these preanalytical methods to process 15 plasma samples from CRPC patients who received anti-androgen therapy prior to the detection of three clinically relevant *AR* gene alterations.

*AR* splice variants, the most common of which is *AR*-V7, are associated with a resistance to *AR*-targeted therapies, as well as to common taxane-based treatments [17]. Our results show that IA-based enrichment of tumor-derived exosomes (Exosomics SoRTEV™ RNA Enrichment Kit) outperforms the total circulating exosomes isolation method in the detection of the *AR*-V7 variant in CRPC plasma samples. As expected, the IA-RNA protocol isolated much less RNA than the generic method, as shown by a 20-fold decrease in the number of copies of the reference gene RNY4 (Figure 1B). However, the sensitivity of detection was increased to 80% (*AR*-V7 detected in 12/15 samples) vs. 53% (8/15 samples), respectively. The *AR*-V7 detection rate after preparation with the generic RNA extraction protocol is consistent with studies that used the same method for *AR*-V7 detection [11]. The increase in sensitivity associated with a higher number of tumor variant copies detected after IA isolation is likely due to an increased signal-to-background ratio that further boosts the low-end sensitivity of dPCR. Indeed, the advantage of tumor exosome enrichment for the detection of *AR*-V7 is appreciated when normalized values (*AR*-V7 vs. RNY4) or *AR*-V7 allelic frequency are compared (Figure 1C and Figure 2C).

**Figure 6:**
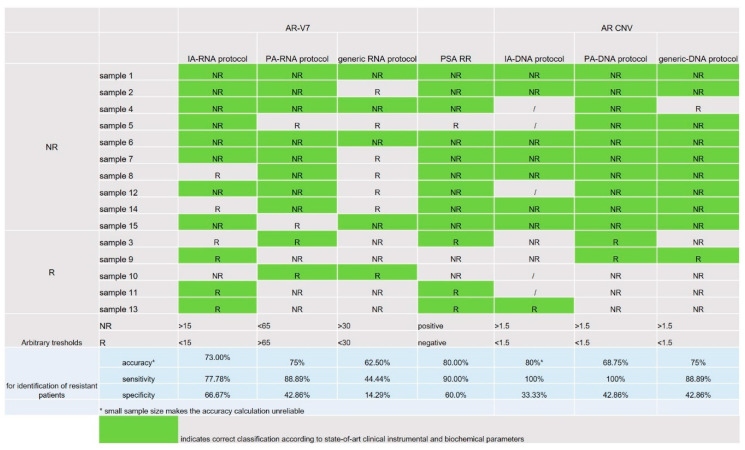
Prediction for a sample to be R or NR based on each single parameter and arbitrary thresholds. In green, the correct correlation with the predicted status according to clinical, instrumental and biochemical parameters.

With the IA enrichment protocol, we were able to distinguish between R and NR patients on the basis of *AR*-V7 results (Figure 2B,D). In our setting, generic protocol fails to show any difference between plasma expression of *AR*-V7 in the two patient groups, although it has been previously reported to correlate well with a patient’s PSA status (PSA response (RR)). A recent meta-analysis reported that relative changes in PSA rather than absolute levels during treatment are an important independent prognostic factor [18]. Indeed, early PSA RR (during first 28 days of treatment) is reported to predict the response to anti-androgen drugs [19]. In our study cohort, classification into NR and R was based on the compendium of standard clinical criteria, which is currently used to guide the treatment decisions (i.e., continue or discontinue treatment). The criteria take into account, but are not limited to, changes in PSA levels. If we compare the PSA RR and the number of *AR*-V7 copies from the total plasma exosomes or the tumor-enriched exosomes, however, the samples processed with the IA-RNA protocol better matched the PSA RR than those processed with the generic isolation protocol (73% vs. 40%), (Figure 6).

The use of plasma exosomes as a source of RNA has already been suggested to improve the detection of CTCs [20,21] as well as whole blood samples [22], although no direct comparison on the same sample set was actually performed. Since *AR*-V7 is a prostate-specific transcript, its detection in whole blood is affected by the contaminating RNA from blood cells that constitutes most of the RNA recovered. Besides directly comparing exosome isolation kits, we also investigated the detection of *AR-*V7 in whole plasma which, unlike whole blood, lacks leucocyte-derived RNA. We detected *AR-V7* in 50% of samples, while reference gene RNY4 and the full-length *AR* transcript were successfully detected in all of them (Figure 1D and Figure 3C). Because whole plasma naturally contains circulating exosomes, the sensitivity of detection of *AR*-V7 does not differ from that observed for total exosomes obtained with a generic solution, but is strikingly lower than the sensitivity observed for enriched tumor exosomes with the IA-RNA protocol.

RNA and mRNA fragments, in particular, are protected and stable within exosomes/EVs, which therefore are privileged and abundant in circulation [23]. Another unique quality of exosomes over freely circulating nucleic acids is that they are released from living cells and therefore their molecular content reflects active tumor growth. Evidence suggests, however, that mRNA fragments can also be protected within circulating DNA-protein complexes, most of which are released from dying cells as DNA–lipoproteins or nucleosomes [8]. The use of preanalytical kits that isolate different samples may help to identify the origin of extracted mRNA targets. For this purpose, we processed the samples with another protocol, namely the PA-RNA protocol, which, by virtue of its combined peptide and charge affinity, co-isolates exosomes of preferential tumor origin and free nucleic acids, particularly DNA. With this kit, we were able to detect *AR*-V7 in 46.6% of the patient samples with a significant increase in copy number detected in the R sample set. The discrepancy observed between the different isolation methods likely stems from the different underlying principles (affinity and/or physio–chemical interactions) by which diverse exosome (sub)populations are harvested along with other plasma components. In this view, the hypothesis is that the IA enrichment protocol preferentially isolates intact exosomes released from living tumor cells, while the peptide-based isolation agent also captures RNA fractions associated to DNA released by dead cells. Therefore, we can expect to observe the increased detection of tumor markers with the IA-RNA protocol in the NR group, and with the PA-RNA protocol in the R group. This exciting hypothesis is indeed supported by our initial data (Figure 3A), but needs further corroboration with larger sample cohorts.

In contrast to previous reports on the association of *AR* splice variants and full-length *AR* expression levels in advanced PCa tissues, we did not observe such correlations, nor significant differences in *AR*-FL expression in exosomes and whole plasma (Figure 3).

The prevalence of *AR* mutations detected in ctDNA has been reported to be consistent with that obtained from matched metastatic tissue (approximately 10–25% of patients) [24]. The *AR* T878A mutation occurs in the ligand binding domain of the androgen receptor and alters the steroid binding properties of the mutated receptor [25]. The frequency of this mutation in CRPC at baseline and that acquired during disease progression does not go beyond 4%, with some reports of the mutation fraction increasing during progression [26]. Its implications for the response to targeted therapeutics, including enzalutamide and abiraterone, are not clear at the moment, with some reports claiming its persistence or possibly conferring resistance to the therapy, while others suggest that its response is maintained in some patients [27,28]. In our study the mutation is detected in nine out of 15 samples (seven out of 10 NR and two out of five R patient samples), with a mutation status that did not coincide in each sample with the different preanalytical protocols (Figure 4). This means that no approach alone was able to reveal mutations in all the samples. Though the exosome-isolation methods did not show overall advantages over the more generic solution for extracting total circulating DNA from plasma, they unambiguously detected T878A in three samples that were negative in cfDNA analysis. This finding is shared by prior observations that exosomes, but not cfDNA, may provide better sensitivity for mutation detection when the allelic density is low and/or predict the occurrence of resistant tumor subclones.

AR copy number amplifications are another mechanism of treatment resistance to common androgen deprivation therapy and can occur in 50% of CRPC patients [29]. The *AR* copy number gain has been associated with disease progression and with a decreased response to abiraterone and enzalutamide [30,31]. *AR* amplification can be detected in cfDNA at the same rate as that obtained from metastatic tissue [25]. Unlike mutations, *AR* amplification is a non-binary variable; therefore, the elevated sensitivity not only in detection, but also in the quantification of the *AR* gain, is likely to be required for a meaningful association to patient outcomes. If we consider a proposed criterion [16], we found *AR* amplification in most of the CRPC samples analysed using either cfDNA or exosome isolation kits (80%) (Figure 5). Although NR and R groups were not clearly distinguished, a gene copy gain is observed in NR samples extracted with all extraction methods, with an (albeit insignificant) increase in the copy numbers detected using the IA-DNA protocol.

The small sample size notwithstanding, our data show that *AR* alterations that were previously reported to be associated with a differential response to *AR* inhibitors as well as to generic treatments, can be detected with a different robustness and sensitivity to different fractions of plasma samples. Moreover, in “qualitative” and unique tumor marks such as *AR*-V7 isoforms (exclusively expressed by prostate epithelium [32]), the enrichment of a subset of circulating exosomes of tumor origin by the IA isolation, rather than the analysis of total plasma exosomes or a whole plasma sample, provides robust and quantitative detection in most samples. In a liquid biopsy setting where low-abundance markers are seen within a complex biofluid sample, the “needle in a haystack” situation can be resolved by “hayremoval” and marker enrichment prior to the use of highly sensitive analytical techniques that are affected by the overall background.

Interestingly, the number of patients with concomitant *AR* alterations, including amplification and mutations, was higher in this study than previously reported [33].

This study supports the idea of a multiparametric approach in liquid biopsy as a next step that would integrate multiple blood-based phenotypes to yield a maximally informative disease profile [34]. The unique claim here is that the term “multiparametric” includes not only multiple markers (typically genes or proteins) but also multiple alterations of the same target (i.e., *AR*) and, ultimately, multiple fractions of the sample that likely reflect the cell or tissue origin of the measured marker. We hereby assessed the feasibility of such an approach and highlighted the advantage of accurate sample fractionation for the detection of shared and unique PCa alterations. Therefore, opting for plasma fractions that are restricted but enriched in tumor exosomes/markers can increase the sensitivity and specificity of downstream analytical methods such as dPCR (used in the current study). The validation of this approach in a larger cohort of both CRPC patients and PCa patients in relation to biochemical progression after primary treatment is warranted to obtain robust evidence of its clinical utility.

## Figures and Tables

**Table 1:** Clinical characteristics of patients (*n* = 13), Samples 2–3 and 11–12 were taken from the same two patients at different time points of treatment. Not available (na).

Sample Code	R/NR	Diagnosis Date	Previous Therapies	Therapy during Withdrawal	Observations	PSA before/after Treatment [ng/mL]
sample 1	NR	2012	Bicalutamide + LHRH analog, Docetaxel + LHRH analog	Abiraterone	metastasis and biochemical progression after abiraterone treatment	177/317
sample 2	NR	2011	Bicalutamide, LHRH analog	Abiraterone	several metastatic loci after abiraterone treatment	546/812
sample 3	R	2011	Bicalutamide, LHRH analog, abiraterone	Docetaxel	steadiness of the disease	812/658
sample 4	NR	2001	Bicalutamide, LHRH analog	Abiraterone	weak instrumental progression after abiraterone treatment	4/151
sample 5	NR	1999	Bicalutamide + LHRH analog, Flutamide + LHRH analog, Zoledronic acid, Docetaxel, Enzalutamide + LHRH analog	Abiraterone	apparent steadiness of illness, but the patient reported a worsening of pain	160/143
sample 6	NR	2003	Bicalutamide + LHRH analog, Abiraterone, Docetaxel + LHRH analog,	Cabaxitaxel	illness progression after cabaxitaxel treatment	42/69
sample 7	NR	2009	Bicalutamide, LHRH analog, Zoledronic acid, Abiraterone, Docetaxel	Enzalutamide	metastasis and biochemical progression after enzalutamide treatment	38/86
sample 8	NR	2004	Bicalutamide, LHRH analog	Abiraterone	instrumental progression after abiraterone treatment	9.3/61.38
sample 9	R	2006	Bicalutamide, Bicalutamide + LHRH analog, LHRH analog, Abiraterone, Docetaxel	Enzalutamide	initial biochemical and instrumental response to enzalutamide	58/125.15
sample 10	R	2008	Bicalutamide + LHRH analog, Zoledronic acid, Bicalutamide, Estramustine	Abiraterone	steadiness of the disease, despite the increase in PSA	7,59/18
sample 11	R	2010	Bicalutamide, LHRH analog, Cyproterone acetate, Abiraterone, Docetaxel, Cabazitaxel	Enzalutamide	initial biochemical response to enzalutamide	266/236
sample 12	NR	2010	Bicalutamide, LHRH analog, Cyproterone acetate, Abiraterone, Docetaxel, Cabazitaxel	Enzalutamide	instrumental and biochemical progression after abiraterone treatment	266/554
sample 13	R	2003	Bicalutamide + LHRH analog, Bicalutamide, Cyproterone acetate,	Enzalutamide	biochemical and instrumental steadiness of the disease	27/0.81
sample 14	NR	2011	LHRH analog, Bicalutamide, Enzalutamide, Docetaxel,	Radiometabolic therapy + LHRH analog	instrumental progression during treatment	82/171
sample 15	NR	2015	bicalutamide + LHRH analog, radiotherapy	LHRH analog	instrumental and biochemical progression during treatment	0.04/26

## Supplementary Materials

Supplementary materials can be found at https://www.mdpi.com/2227-9059/8/5/131/s1.
