# Supplementary material for: When Less Is More: Specific Capture and Analysis of Tumor Exosomes in Plasma Increases the Sensitivity of Liquid Biopsy for Comprehensive Detection of Multiple Androgen Receptor Phenotypes in Advanced Prostate Cancer Patients
Source: Biomedicines. 2020 May 22;8(5):131. doi: 10.3390/biomedicines8050131 (PMC7277361; doi:10.3390/biomedicines8050131)
Supplement: Supplementary file 1 [file biomedicines-08-00131-s001.pdf]

biomedicines

S2. Supplementary Material and Methods

S.2.1 Cell cultures and exosome isolation

Cell supernatants from Hek293, ASPC1 and 22RV1 were collected and were pre-cleared with three centrifugation steps (300g 10 min +4°C, 1200g 20 minutes +4 °C, 10000g 30 min +4 °C) and concentrated to a final volume of 10 ml with Amicon 100K filter (Merck). Extracellular vesicles were purified from conditioned supernatants cells using SEC Columns qEV 10-35 (Izon). ELISA assay for CD9 (Exbio) and microBCA assay (Thermo Fischer Scientific) were used to identify the fractions positive for EVs and containing less protein contaminants. These fractions were pooled and concentrated to a final volume of 200-500 ul with Amicon 100K filter. Noteworthy, the purity and size distribution of the isolated material was confirmed by NTA (Nanosight tracking analysis) and BCA assay (Thermo Fischer Scientific). Namely the size of the vesicles ranged between 70 and 150 nm, with a dominant peak at 90-100 nm which is compatible with exosome-like vesicles. The purity, expressed as the ratio between particle number (measured at NTA) and total protein concentration (BCA), was higher than 2E+09, confirming high purity of vesicle preparation [35].

S.2.2 FACS analysis of EV binding antibodies

Purified EVs have been absorbed on the surface of 4 micron latex beads and incubated with either SoRTEV (Exosomics SpA) or aCD63 antibody (ExBio) at concentrations of 5 ug/ml. Isotype IgG1 antibody (ExBio) at the same concentration was used as a control. FACS analysis was performed on BD FACSCanto II.

S.2.3. Western Blot assays

Upon the incubation of sample with SoRTEV beads, the beads based pelleted material (see 2.2.) was resuspended with PBS and Laemmli sample buffer 4X containing β-mercaptoethanol (Bio-Rad), denatured at 95°C and loaded on 4-20% Mini-PROTEAN gel (Biorad). The gel was transferred on nitrocellulose membrane using Trans-Blot Turbo Transfer System and Trans-Blot Turbo Mini 0.2 µm Nitrocellulose Transfer Pack (Bio-Rad). Alix target was detected using Alix C-11 antibody (Santa Cruz Biotechnology) 1:500 in PBS-Milk 0.5% and mouse secondary antibody (Bio-Rad) 1:20000 in PBS-Milk 0.5%. SuperSignal™ West Femto Maximum Sensitivity Substrate (Thermo Fisher Scientific) was used for the detection of the signals. Image was acquired by Image Quant LAS 500 (GE).

S.2.4. TEM analysis

Pellet obtained after SoRTEV™ isolation from plasma samples, was washed with SoRTEV kit provided washing buffer and resuspended in 100 µl of PBS. Later, 3-µl drop of sample was placed on a 300 mesh formvar coated copper grid for 2 minutes. Blotted the excess with filter paper, the grid was negatively stained with 1% aqueous Uranyl Acetate for 30 seconds and analyzed using a FEI Tecnai G2 Spirit TEM operating at 120 kV equipped with an EMSIS Veleta CCD camera.

S.2.5. Droplet digital PCR analysis of tumor marker enrichment in EVs isolated from cell models and cancer patient samples

For spike-in analyses, a defined quantity of exosomes was added to plasma samples of healty individuals. Exosome isolation was performed with protocols described in Material and Methods section (see 2.2.) and RNA/DNA was extracted with column-based workflow. RT-qPCR was performed on CFX Connect Real-Time PCR Detection System (Biorad) with iTaq™ Universal Probes One-Step Kit (Biorad). Each reaction consisted of 1X one-step reaction mix, 0.625 µl iScript™ reverse transcriptase, 1.25 µl primers (10 µM), 0.625 fluorescet probe (10 µM), 2.5 µl extracted RNA for a total of 25 µl (primer/probes for ARV7 already described in the paper and reported by Morandi et al. [36] for KRAS G12D) After mixing, each reaction was loaded in triplicate on a 96-well PCR plate and the following qPCR program was launched: 50°C for 10', 95°C for 3’, 40 cycles at 95°C for 15'' and 60°C for 30’’. Data analysis was performed with $2^{-\Delta\Delta Ct}$ method, using Y4 as internal control. Allele-specific

PCR on BRAF V600E was a modified version of the allele-specific locked nucleic acid PCR [36]. Briefly, each qPCR reaction included 10 µl of DNA eluate or 10ng of genomic DNA as positive control (Horizon Discovery), 1X SsoAdvanced Universal Probes Mastermix (Biorad; US), 0.625 µl of primers (10 µM) and 0.3125 µl of fluorescent probe (10 µM) in a total volume of 25 µl. After careful mixing, each reaction was loaded in triplicate on a 96-well PCR plate and the following qPCR program was launched: 95°C for 3', 40 cycles at 95°C for 5'' and 60°C for 30''. $BRAF^{V600E}$ and $BRAF^{WT}$ recovery was expressed as Ct value, represented on the Y axis in decreasing order.

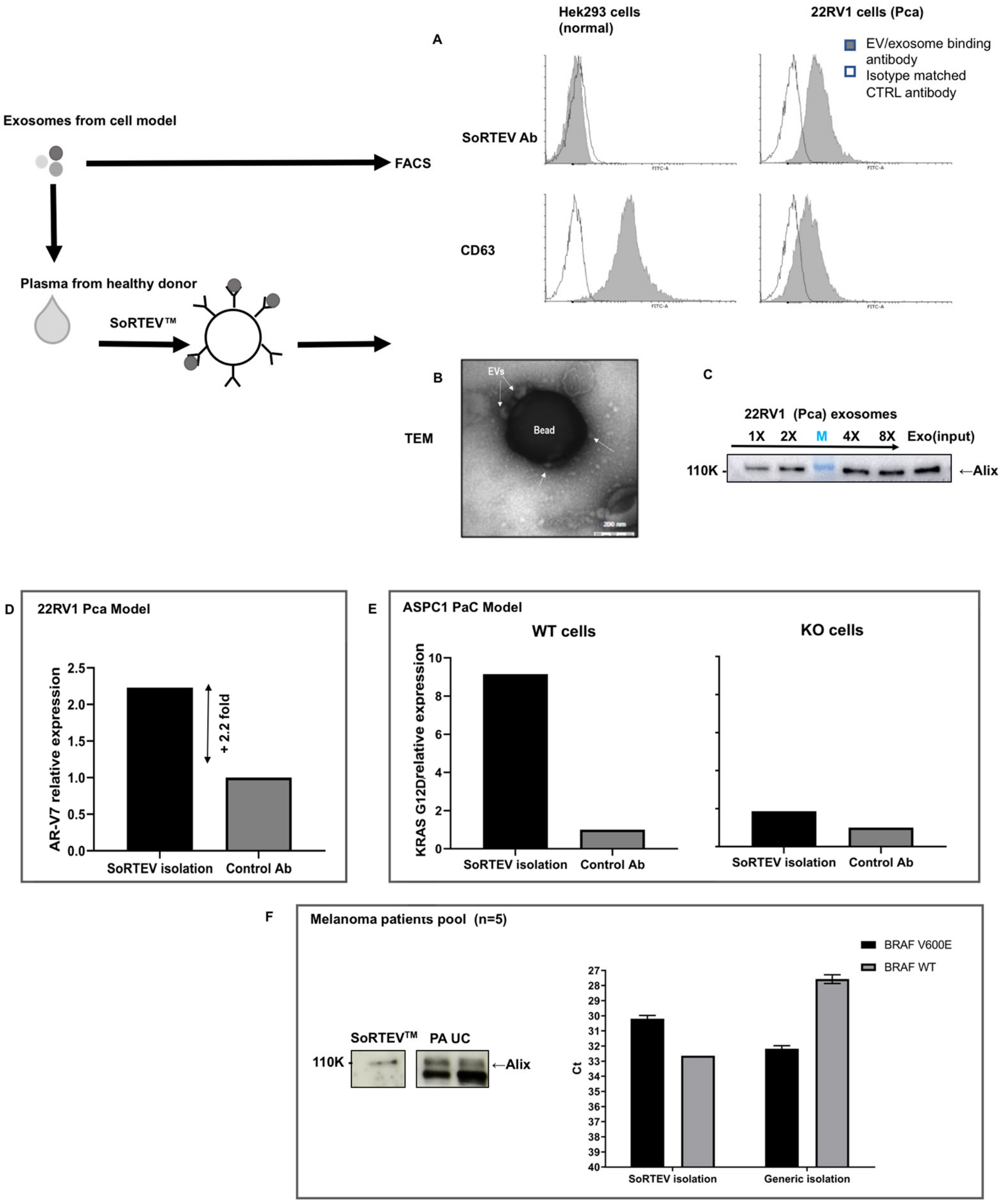

Figure S1. (**A**) FACS analysis of CD63 and SoRTEV mAb binding exosomes isolated from PCa cell line (22RV1) and from non-cancer cell line (Hek) conditioned medium. (**B**) TEM Uranyl Acetate counterstaining of SoRTEV beads incubated with at least 1E+09 Particles of 22RV1 tumor exosomes spiked in plasma. (**C**) Western Blot analysis for Alix expression of SoRTEV pellets after recovery of 22RV1 exosomes (at least 1E+09 Particles) spiked into 1 ml of plasma. Increased concentration of immunobeads have been used and the highest concentration was adopted for the prototype SoRTEV kit used in the present study. The

exosome input (1E+09 Particles) is loaded on the lane to the right. (**D**) Exosomes from PCa cell line (22RV1) were spiked in 1 ml of healthy human plasma. Exosomes isolation was performed with SoRTEV mAb and isotype-matched mAb. RT-qPCR analysis showed an enrichment of ARV7 mutation with SoRTEV mAb respect to control mAb. (**E**) The same comparison was performed by spiking exosomes of pancreatic tumor cell line (ASPC1) in healthy plasma. The point mutation KRAS G12D was detected by RT-qPCR with great enrichment with SoRTEV mAb. The same trend was not observed with KO cells for the target of interest. (**F**) On the left, Western Blot of EVs isolated with SoRTEV protocol from plasma of metastatic melanoma patient, compared to generic isolation methods (PA: peptide affinity; UC: ultracentrifuge). On the right, SoRTEV isolation was performed on pooled plasma from a cohort of BRAF-V600E positive melanoma patients (n=5) and, in parallel isolation with ME-kit (PA, peptide affinity). After DNA extraction, qPCR was used to detect BRAF V600E and BRAF WT.

Supplementary References

35. Webber, J.; Clayton, A. How pure are your vesicles? *J.Extracell.Vesicles* **2013,** 10, 2.. doi: 10.3402/jev.v2i0.19861

36**.** Morandi, L.; de Blase, D.; Visani, M.; Cesari, V.; De Maglio, G.; Pizzolitto, S.; Pession, A.; Tallini, G. Allele specific locked nucleic acid quantitative PCR (ASLNAqPCR): an accurate and cost-effective assay to diagnose and quantify KRAS and BRAF mutation. *PlosOne* **2012**,7,e36084. doi: 10.1371/journal.pone.0036084.
